# A comparative analysis of artificial intelligence generated 3d facial models from 2D photos: A procrustes-based validation of landmark accuracy in Orthodontics

**DOI:** 10.1053/j.sodo.2025.10.014

**Published:** 2025-10-29

**Authors:** Jonas Bianchi, Lorena Wilka, Gabriel Bravo Vallejo, Felicia Miranda, Camila Massaro, Lucia Cevidanes, Heesoo Oh

**Affiliations:** a Department of Orthodontics, University of the Pacific, Arthur A. Dugoni School of Dentistry, San Francisco, CA USA; b Department of Orthodontics, Bauru Dental School, University of São Paulo, Bauru, SP Brazil; c Department of Orthodontics, Hospital of Rehabilitation of Craniofacial Anomalies, University of São Paulo, São Paulo, Bauru, SP Brazil; d Department of Orthodontics, The University of North Carolina, Chapel Hill, NC USA

**Keywords:** Facial landmarks, Soft tissue morphology, 3D facial imaging, Artificial intelligence, Facial scanner, facial analysis

## Abstract

**Objectives:**

The primary aim of this study was to evaluate the accuracy and precision of a deep learning artificial intelligence (AI) model in reconstructing 3D facial geometry from a single 2D photograph. The study specifically measured the coordinate differences of human-placed facial landmarks on the AI-generated models against those placed on 3D models from a gold-standard scanner (Bellus 3D). Secondary objectives included identifying dimensional variations (X, Y, Z) and systematic biases in the AI reconstructions

**Methods:**

This was a retrospective comparative study using a sample of 47 anonymized patient records from the University of the Pacific database. For each patient, a 3D facial model was generated from a 2D photograph using the 3DDFA-V2 deep learning model. These AI-generated models were compared to reference models captured on the same day using a Bellus 3D Scanner. Two trained observers identified a set of facial landmarks on both the AI-generated and Bellus 3D models. The coordinate data were normalized using Procrustes analysis to compare shape and landmark position independent of scale and orientation. The differences in landmark coordinates between the two model types were then statistically analyzed.

**Results:**

The analysis revealed that human landmark identification on the AI-generated 3D models reconstructed from 2D photos showed consistently lower variability compared to the gold-standard scans, suggesting that those models may lead to more repeatable measurements. The greatest overall deviations (Euclidean error) were found in landmarks associated with the lateral and inferior borders of the face, such as Right Gonion (RGo), Trichion (Tr), and Left Gonion (LGo). Quantitatively, the mean Procrustes-normalized error across all landmarks was 0.19 ± 0.08 units (range: 0.08–0.45), corresponding approximately to 0.2 mm of proportional deviation when referenced to the 1:1 scale Bellus3D models. While the AI-generated model demonstrated high fidelity, systematic biases were observed mainly as inferior displacements of key landmarks, including Menton (−0.35 units) and Pogonion (−0.30 units), reflecting a consistent downward shift in AI-based reconstruction

**Conclusions:**

The 3DDFA-V2 deep learning application demonstrated in this study is a promising low-cost tool for generating 3D facial models from 2D photographs with a high degree of precision in landmark placement. While the AI models facilitated more repeatable landmarking, they also exhibited some systematic biases. These findings have clinical implications for the use AI-generated 3D models in diagnosis and treatment planning, highlighting areas where further refinement of the AI algorithm is needed.

## Introduction

The comprehensive analysis of facial soft tissue has been widely done in modern orthodontic and dentofacial orthopedic diagnosis and treatment planning. While traditional assessments focused primarily on skeletal and dental relationships, the soft tissue ultimately dictates facial esthetics and patient satisfaction. Key clinical evaluations, including the assessment of facial proportions, profile convexity, lip fullness, and the projection of the chin and maxilla, are dependent on accurate soft tissue measurements. Metrics such as the nasolabial angle, mentolabial angle, and the relationship of the lips to Ricketts’ E-line are critical for establishing a harmonious facial balance and predicting the aesthetic outcome of orthodontic interventions.^1–3^

Historically, clinical facial analysis has relied on two-dimensional (2D) photography, which is limited by projection and distortion errors. Three-dimensional (3D) imaging technologies, including stereophotogrammetry and structured light scanning, have overcome many of these limitations by providing accurate, repeatable, and comprehensive surface data that allow reliable assessment of facial symmetry and proportions.^4–6^

However, this speed and accuracy come at a significant financial cost, and the technology is limited in its ability to capture deep concavities on the facial surface. Structured light scanners, which project a pattern of light onto the face to calculate its geometry, offer a more cost-effective alternative. Their primary problems are a scan time of several seconds, during which the patient must remain perfectly still, and a sensitivity to strong ambient light that can degrade scan quality. The high cost and technical requirements of these systems present a significant barrier to larger adoption in clinical settings.^7,8^ Stereophotogrammetry systems can cost anywhere between $30,000 to $80,000 and capture images in under one second, ideal for rapid orthodontic facial scans, while structured light scanners range $10,000–$40,000.

This clinical and financial challenge creates a need for a low-cost, accessible, and accurate solution for 3D facial reconstruction. The rapid advancement of artificial intelligence (AI), particularly in the field of deep learning, presents a novel and promising pathway to address this need.^9–11^ Advanced AI models, such as the 3DDFA-V2 model^12^ used in this study, are now capable of generating a detailed 3D facial mesh from a single 2D photograph by predicting parameters for a 3D Morphable Model (3DMM). While these AI-based reconstruction tools are becoming increasingly accessible, most have been trained and validated on general facial datasets that lack representation of orthodontic populations and clinical variability. As a result, their performance in capturing clinically relevant soft-tissue landmarks and facial proportions remains uncertain.

Therefore, purpose of this study was to quantify the accuracy and precision of a deep learning AI model in reconstructing 3D facial geometry by comparing the coordinates of human-annotated landmarks on AI-generated 3D models reconstructed from 2D photos to those on models from a Bellus 3D Scanner. The null hypothesis was that there are no statistically significant differences in the error distributions for specific facial landmarks identified on the 3D models generated from 2D photos by the AI algorithm when compared to the gold-standard models obtained from the 3D scanner. Procrustes analysis was selected as the validation metric because it allows comparison of shape configurations independent of translation, rotation, or scale, making it well-suited for assessing geometric similarity between 3D facial models.

## Materials & methods

### Study design and ethical approval

This was a retrospective comparative study. The protocol was reviewed and approved by the Institutional Review Board (IRB) at the University of the Pacific (IRB2022–125). The study sample consisted of records from 47 patients obtained from the clinical database of the University of the Pacific Arthur A. Dugoni School of Dentistry. For each subject, the initial 2D facial photograph and the initial 3D facial scan were used as the source data for the analysis. The inclusion criteria were that both the 2D photograph and the 3D scan for each patient were acquired on the same day to ensure consistency. During both photographic and 3D scanning sessions, subjects were instructed to maintain a neutral facial expression with lips gently relaxed and teeth in light occlusion. Inclusion criteria required absence of facial deformity and no orthodontic appliances at the time of image capture. All 2D photographs were taken under similar conditions using a fixed camera-to-subject distance of approximately 1.5 m, uniform frontal and back lighting, and a DSLR camera. However, one limitation of this study is that there were slight variations in camera brand and exposure settings. The sample comprised 47 individuals (21 females, 26 males) with a mean age of 21.7 ± 11.1 years (range: 9.1–59.5 years).

### 3D Model generation

Two distinct types of 3D facial models were generated for each subject: a gold-standard model from a direct 3D scan (Bellus 3D) and an experimental model: AI-generated 3D models reconstructed from 2D photos using an AI algorithm called 3DDFA-V2 model.^12^

### Gold-Standard models: Bellus 3D scanner

The reference or “gold-standard” 3D facial models were acquired using the Bellus3D facial scanning system, reconstructed and exported to .obj using the native software Bellus 3D Dental Pro (discontinued). This system utilizes stereophotogrammetry to produce high-resolution and texturally accurate color models of the facial surface, serving as the baseline for comparison in this study.^13^

### AI-generated 3D models reconstructed from 2D photos: the 3DDFA-V2 deep learning framework

The experimental 3D models were reconstructed from 2D photographs using a deep learning model known as 3DDFA-V2 (3D Dense Face Alignment, Version 2).^12^ Instead of directly regressing the coordinates of tens of thousands of vertices, the 3DDFA-V2 model employs a more efficient and stable method based on a 3D Morphable Model (3DMM). A 3DMM is a powerful statistical model that can generate a wide range of realistic 3D faces by changing a small set of parameters that control shape, expression, and pose.^14,15^ The lightweight network architecture of the 3DDFA-V2 model is trained to analyze a 2D image and predict this small set of 3DMM parameters. The model was trained on the 300W-LP dataset, an extensive collection of 61,225 facial samples across various poses (https://github.com/cleardusk/3DDFA_V2). The code was downloaded and adapted for local processing. The AI reconstruction workflow, illustrated in Fig. 1, proceeds through a series of automated steps. The final AI-created models and Bellus 3D scanner models are shown in Fig. 2. All 3D reconstructions were performed on a workstation equipped with an NVIDIA RTX 3080 GPU (10 GB VRAM), Intel Core i9 processor, and 64 GB RAM. Each model was processed in approximately 3–5 s.

### Initial imaging processing and landmark identification and reliability

Prior to analysis, all Bellus 3D facial scans were spatially oriented using a standardized procedure to ensure consistent alignment of reference axes. This was performed in Python (VS Code environment) using the VTK library’s transformation utilities (e.g., vtkTransform and vtkLandmarkTransform). The analysis was performed by two orthodontist examiners (L.W. and G.B.), who digitally identified and marked 25 anthropometric landmarks (14 bilateral and 11 midline) on each 3D facial image, as seen in the Fig. 3. For each landmark, the 3D-Slicer^16^ software recorded the coordinates in the three spatial axes (x, y, z) and Euclidean distance. The full list of these landmarks, their abbreviations, and their precise anatomical definitions are provided in Table 1. Intra-examiner reliability exceeded 0.90 for all coordinates, and inter-examiner intraclass correlation coefficients ranged from 0.89 to 0.96. To minimize random variation, the mean coordinate position of both examiners was used for subsequent analysis, as this approach provides a robust estimate of the true landmark position while preserving reproducibility.

The workflow was as follows: the 3D images were obtained using Bellus 3D Scanner and AI-generated models. The Bellus 3D scanner produced files in OBJ and JPG formats, whereas the AI generated files in PLY format, already containing color information, eliminating the need for additional processing steps. All images were imported into 3D Slicer, an open-source platform for medical image visualization and analysis. For OBJ/JPG files: Files were uploaded into the program; The Surface module was opened, and the Texture model option was selected; Under “save color information as point data,” the RGB vector was selected and applied, enabling textured 3D model visualization; For PLY files, the colored model was automatically recognized without requiring additional steps.; Anatomical landmarks were manually placed using the Markups module, which allows three-dimensional reference point creation on the facial reconstruction; The evaluation was performed exclusively in 3D reconstruction view, without the use of multiplanar slices; Landmarks were saved in landmark file (.mrk.json) format, allowing subsequent export for quantitative analysis. Coordinates were extracted and recorded in Microsoft Excel, (Microsoft Corporation, version 2508, Redmond, WA, USA) for statistical processing and using the Python language.

### Data normalization and statistical analysis

To compare the landmark configurations from the AI and Bellus3D models, Generalized Procrustes Analysis^17^ was employed. This method standardizes the data by computationally overlaying the two sets of landmarks for each patient and removing non-shape variations related to translation (position), scale (size), and rotation (orientation). This normalization process isolates the true shape differences, allowing for a direct comparison of landmark positions in a common coordinate system. Following Procrustes alignment, the positional error for each landmark was calculated as the coordinate of the AI-placed landmark minus the coordinate of the gold-standard landmark (Error = AI - Bellus). This calculation was performed for the three spatial dimensions: transverse (X-axis), superior-inferior (Y-axis), and anterior-posterior (Z-axis). The overall error magnitude, regardless of direction, was calculated as the 3D Euclidean distance. All error measurements are reported in Procrustes-normalized units, which represent proportional shape deviation rather than absolute distance. Subsequent statistical analyses were performed to evaluate the distribution, mean, and variability of these errors. For overall comparison, the mean Procrustes-normalized error across all analyzed landmarks was 0.19 ± 0.08 units (range: 0.08–0.45), equivalent to approximately 0.2 mm of proportional deviation when referenced to the 1:1 scale Bellus3D models. All analyses were performed in SPSS (Version 28) and Python (Version 3.8.18). Normality of coordinate error distributions was assessed using the Shapiro–Wilk test. A significance threshold of *p* < 0.05 was adopted, with Bonferroni correction applied to multiple comparisons.

As a clinical reference, the Procrustes-normalized values can be approximately related to millimeter equivalents by referencing the 1:1 scale Bellus 3D models, where 0.05 normalized units correspond to roughly 0.05 mm of proportional deviation. This conversion was provided solely to aid clinical interpretation; all analyses were performed using normalized values exclusively.

## Results

### Intra-examiner reliability and landmark placement errors

The reliability of the manual landmark identification process was assessed prior to the main analysis. The intra-examiner reliability was found to be high, with an Interclass Correlation Coefficient (ICC) above 0.90 for the majority of the landmark positions evaluated. The positional errors, calculated as the difference between landmark coordinates on the AI-generated models and the gold-standard Bellus 3D models after Procrustes alignment (Error = AI - Bellus), were analyzed across three dimensions (X, Y, Z) as shown in Figs. 4–6, respectively, and a total 3D Euclidean distance, shown in Fig. 7. A global overview of the error distributions across all landmarks is presented in these violin plots. They showed that the mean errors for the transverse (X), superior-inferior (Y), and anterior-posterior (Z) axes are centered near zero. However, the plots also indicate a wide distribution of errors, representing the variability in landmark placement between the two model types. The error is reported in “Procrustes-normalized error” rather than absolute measurements such as millimeters. This occurs due to the procrustes normalization,^17^ which ensures that the results are independent of the subject’s individual facial size, allowing for a better comparison of accuracy across the entire dataset. Therefore, a value of 0.05 on the vertical axis represents a localization error that is equivalent to 5 % of the standard reference dimension used for normalization.

### Analysis of systematic error (Bias)

To investigate systematic bias, 95 % confidence intervals for the mean error of each landmark were calculated and plotted for each spatial axis, as shown in Fig. 8. A systematic bias is indicated when the 95 % confidence interval for a landmark does not include zero. Transverse (X-axis) Error: For most landmarks, the 95 % confidence intervals for mean transverse error crossed the zero line, indicating no statistically significant systematic bias. A notable exception was the Left Gonion (LGo), which demonstrated a consistent negative mean error, suggesting the AI model tended to place this landmark slightly more to the left than the gold-standard method. Superior-Inferior (Y-axis) Error: A systematic bias in the vertical dimension was observed for several landmarks. A pattern of negative mean errors was found for landmarks including Left Pupil (LPup), Menton (Me), and Pogonion (Pog), indicating that the AI model systematically placed these landmarks in a more inferior position compared to the gold-standard scans. Anterior-Posterior (Z-axis) Error: The systematic bias in the A-P dimension was mixed and landmark-specific. For example, a significant positive mean error was observed for Glabella (Gl), indicating a slightly more anterior placement by the AI model, while a significant negative mean error for Trichion (Tr) indicated a more posterior placement.

To further evaluate the distribution of AI reconstruction accuracy, errors were examined for each individual landmark, as shown in Fig. 9. The Left Gonion (LGo) and Right Gonion (RGo) were excluded from this analysis because the AI model consistently failed to reconstruct these boundaries of the face. For the remaining landmarks, the Root Mean Square Error (RMSE) calculated as the square root of the mean squared deviation was used to quantify accuracy. Fig. 9 presents the RMSE values by landmark. It is clear that while the gold-standard scans exhibited greater spread in landmarking (likely reflecting the complexity of real-world surface anatomy), the AI reconstructions produced lower variability, suggesting that human raters were more consistent when working with the smoother, photo-based surface

### Limitations

This study has some limitations. The most significant was the issue of image scaling. A consistent, absolute scale could not be maintained between the AI-reconstructed models and the gold-standard scans, which necessitated the use of Procrustes analysis to normalize for size and the use of % instead of mm for comparison. While this is a standard method for shape comparison, it means that all error data is presented in proportional, normalized units rather than absolute millimeters, which can complicate direct clinical interpretation of error magnitudes. Furthermore, the scope of this investigation was limited to the comparison of a single AI model against a single type of 3D scanner, the Bellus 3D Although the Bellus 3D scanner used as the reference standard has been discontinued, its structured-light technology and geometric accuracy are comparable to those of other contemporary 3D surface imaging systems, such as stereophotogrammetry and static structured-light scanners. Therefore, the present validation results are expected to generalize to currently available similar technologies, but not to other 2D-to-3D reconstruction algorithms or alternative gold-standard imaging systems.

## Discussion

This study aimed to evaluate the application of a novel, low-cost deep learning model (3DDFA-V2)^18^ for 3D facial reconstruction from 2D photographs, using a gold-standard 3D scanner as the reference. The primary finding was that human-placed landmarks on the AI-generated models exhibited lower variability and therefore higher precision than those placed on the gold-standard scans. The higher repeatability of landmark placement on the AI-reconstructed models suggests that the AI’s process of generating a 3D mesh from a single 2D image results in a computationally smoother facial topography. The gold-standard scans, in contrast, capture every surface detail, which can create ambiguity for a human operator attempting to pinpoint a single coordinate for a landmark. Fuyamada et al.^19^ (2011) showed that using plane-specific definitions for CBCT landmarks improved reproducibility compared with traditional cephalometric definitions, independent of evaluator experience. In addition, the smoother AI surface may provide a less ambiguous target, leading to more consistent placement. Such AI models may offer an unexpected advantage over more detailed but “noisier” gold-standard data, which itself is known to have baseline variability in landmark identification, as established by Ayoub et al.^20^ (2013).

While precise, the AI models were not without inaccuracies. The systematic errors observed suggest inherent biases in the reconstruction algorithm, a finding that aligns with the broader literature on 2D-to-3D conversion. Recent studies by Mao et al.^21^ (2024), Morales et al.,^22^ (2021), and Zhou et al.,^23^ (2024) highlights that predictable error patterns are a common challenge across deep learning models for facial reconstruction. In our results, the consistent inferior placement of some landmarks like Menton and Pogonion could stem from how the model interprets shadowing in a 2D photograph or from biases within its training data. As the model was trained on its own dataset, it is expected that there are limitations from the dataset’s own creation process, as discussed by Zhu et al. (2016).^24^

The largest errors were found at the facial periphery, particularly the gonial angles (RGo, LGo) and the hairline (Tr). This finding was expected, as these regions are poorly defined in a frontal 2D photograph and rely on the algorithm to define the geometry from the profile. This limitation reflects a fundamental challenge in 3D craniofacial analysis. Prior studies of landmark reliability on stereophotogrammetry and CBCT data have consistently shown that peripheral landmarks, such as gonion and condyle, are among the least reliable for manual identification. For example, Baysal et al.^25^ (2016) demonstrated that while most soft tissue landmarks placed on 3D stereophotogrammetric images were reproducible within 1 mm, variability increased at peripheral points.

Overall, the present results demonstrate high precision and repeatability of AI-generated facial reconstructions, reflecting the robustness of the 3DDFA-V2 algorithm in maintaining consistent landmark geometry. Nonetheless, systematic biases were observed in certain regions, likely associated with the intrinsic smoothness of AI-generated surfaces and the absence of real-surface texture irregularities that typically characterize clinical scans. Similar findings have been reported in recent dental and orthodontic AI studies,^13,21,26^ which emphasize that model training on general facial datasets may underrepresent morphological variations relevant to orthodontics. These findings highlight both the promise and current limitations of AI-based 3D reconstruction, underscoring the need for domain-specific datasets and validation frameworks tailored to orthodontic populations.

These results have direct clinical and research implications. The AI model shows significant promise as a low-cost, radiation-free, and highly accessible tool for 3D facial analysis, especially for assessing midline facial structures and overall facial harmony, where high repeatability is beneficial. However, our findings urge caution when using these models for treatments that demand sub-millimeter accuracy at the facial periphery, such as surgical planning for orthognathic cases. As highlighted by Serafin et al.^26^ (2023) in their systematic review and meta-analysis of automated 3D cephalometric landmarking, mean landmarking errors across deep learning systems still average around 2–3 mm, with the greatest errors seen at peripheral site. From a clinical standpoint, AI-based 3D facial reconstruction offers promising applications in orthodontics, including digital records, treatment monitoring, and smile or growth analysis. Future studies should further correlate proportional (Procrustes-normalized) errors with millimeter-based measurements to establish direct clinical thresholds for acceptable accuracy

## Conclusion

This study demonstrated that a deep learning AI model can reconstruct 3D facial models from 2D photographs with high precision when compared to a gold-standard 3D scanner. The AI-generated models, likely due to their computationally smoother surfaces, permitted more repeatable landmark identification by human operators and showed consistently lower variability than the gold-standard scans, which exhibited greater random error from their real-world complexity. However, this enhanced precision was accompanied by systematic biases in the AI models, including shifts in soft tissue landmark positions, indicating limitations in the model’s ability to accurately predict 3D depth from a 2D image. Therefore, while this AI-based method represents a promising and highly accessible tool for 3D facial analysis, its current limitations in accuracy warrant clinical caution, especially for applications requiring high fidelity at the facial analysis.

## Figures and Tables

**Fig. 1.**
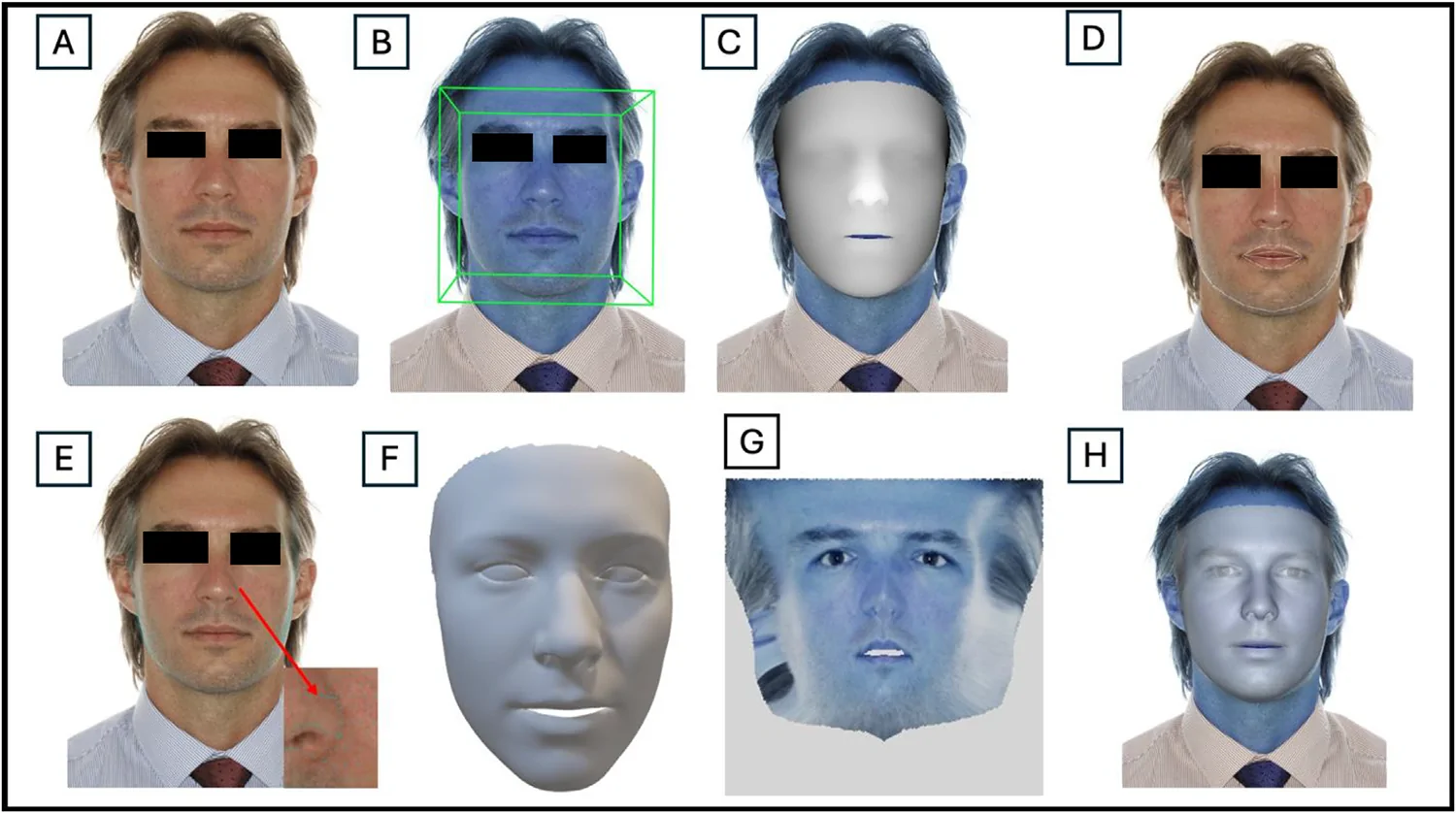
AI-Generated 3D facial mesh overview. A) Input Image: The process begins with a single 2D frontal photograph as input; B) Pose Estimation: The algorithm first detects the face and estimates its 3D orientation (pose) to normalize its position in space; C) Depth Map and Landmark Prediction: The model then predicts a depth map to provide the z-coordinate for the facial surface and identifies both a sparse and a dense set of 2D landmarks as seen in D; E)Mesh Generation: This information is integrated to generate a 3D dense point cloud, which is then converted into a non-textured 3D mesh surface and saved as a PLY or OBJ file as shown in F; G) Texturing and Final Output: a 2D texture map is created from the original photo and wrapped around the 3D mesh to produce the final textured 3D model. H) The final result, without application of the texture.

**Fig. 2.**
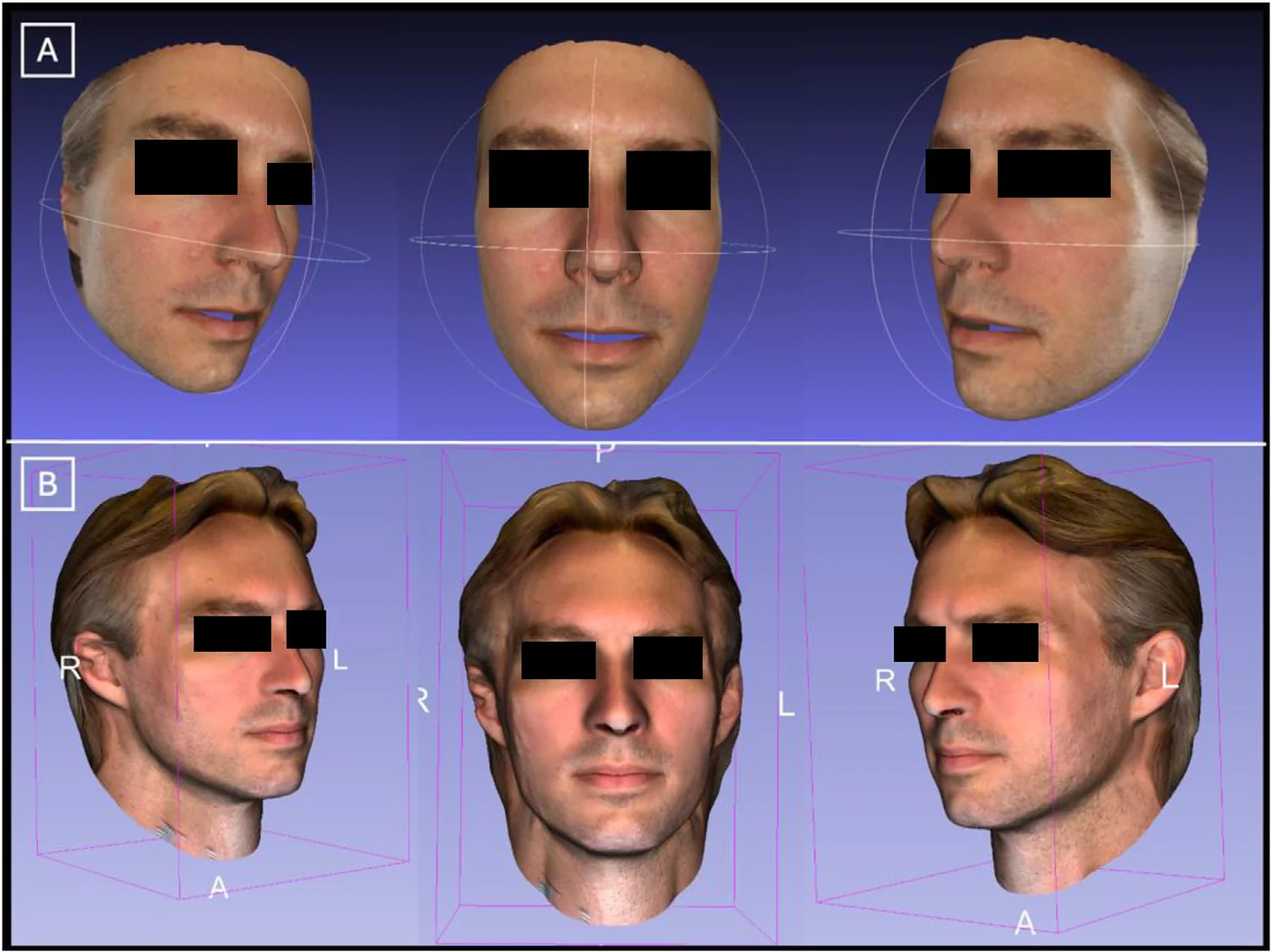
Qualitative Comparison of 3D Facial Models from (A) AI Reconstruction and (B) a Bellus 3D Scanner.

**Fig. 3.**
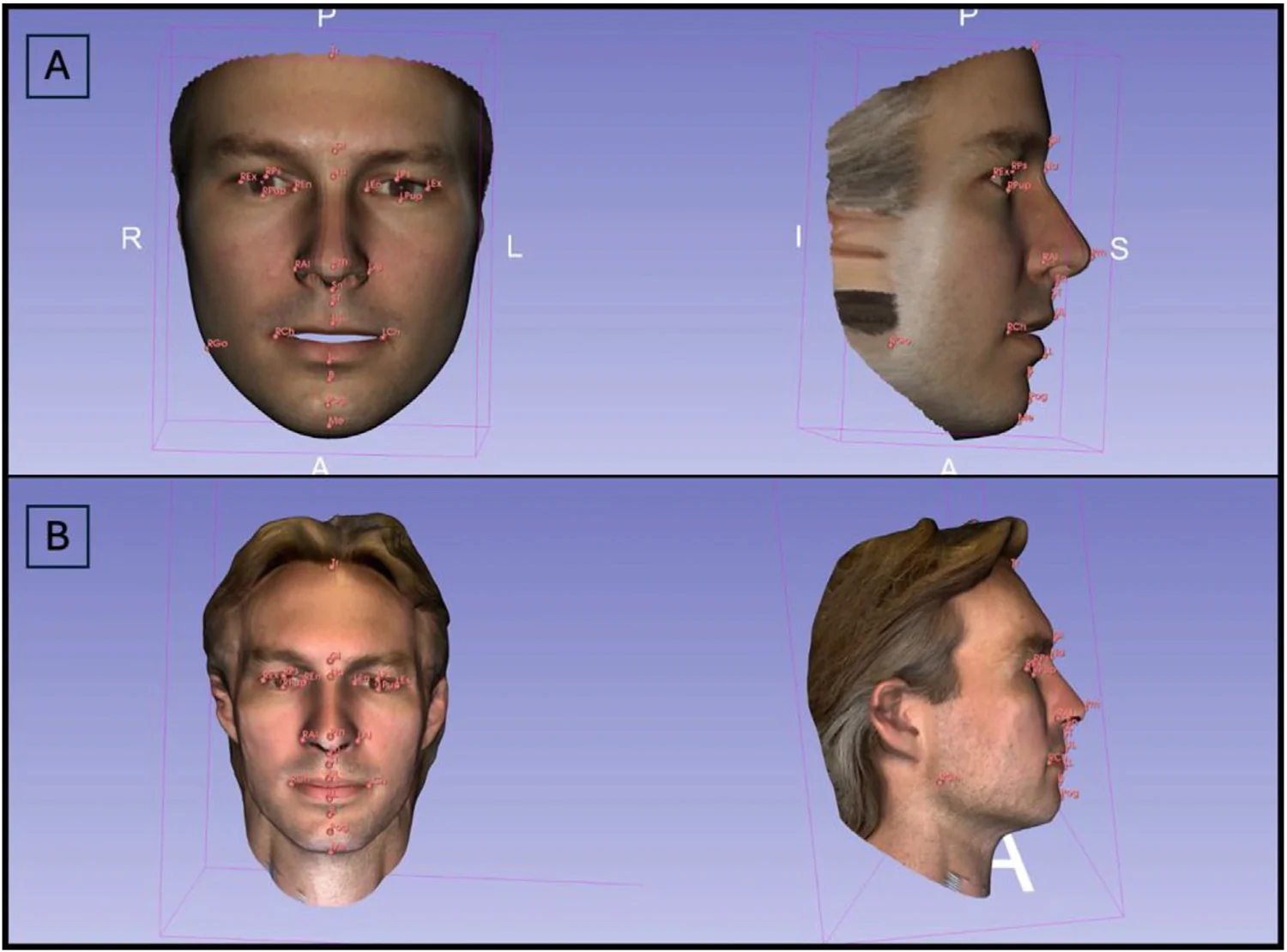
Landmarks placement and Comparison of 3D Facial Models from (A) AI Reconstruction and (B) a Bellus 3D Scanner.

**Fig. 4.**
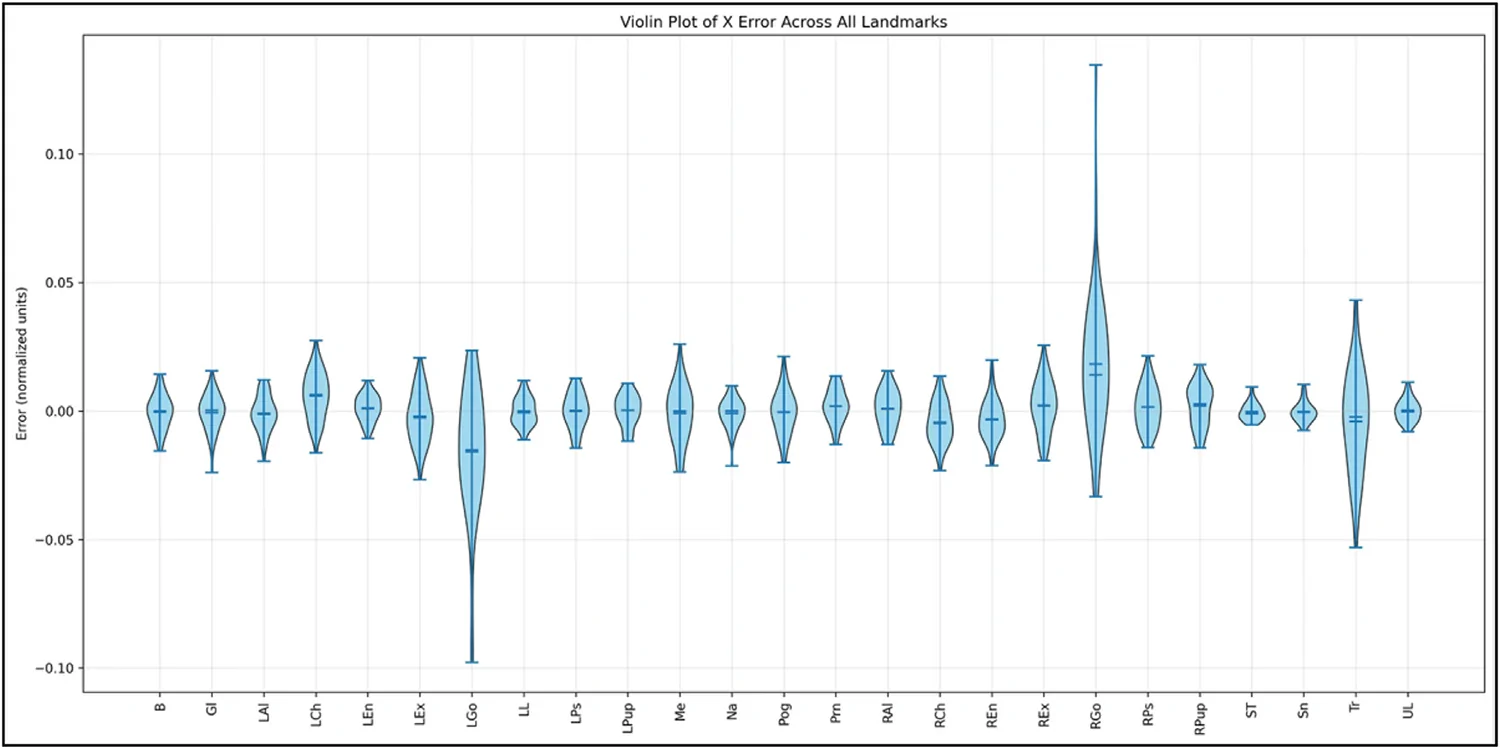
Violin Plots of Transverse (X-axis) Error by Landmark.

**Fig. 5.**
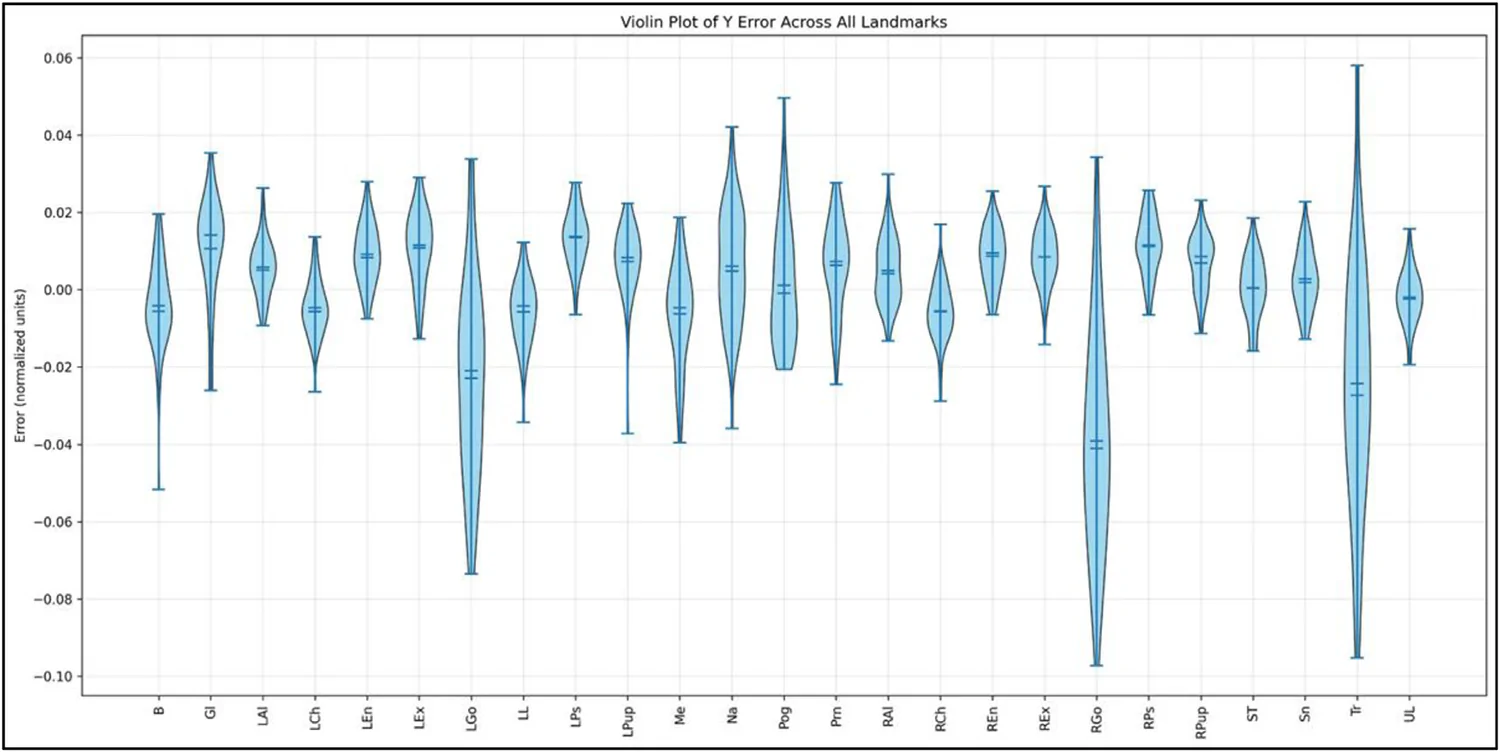
Violin Plots of Superior-Inferior (Y-axis) Error by Landmark.

**Fig. 6.**
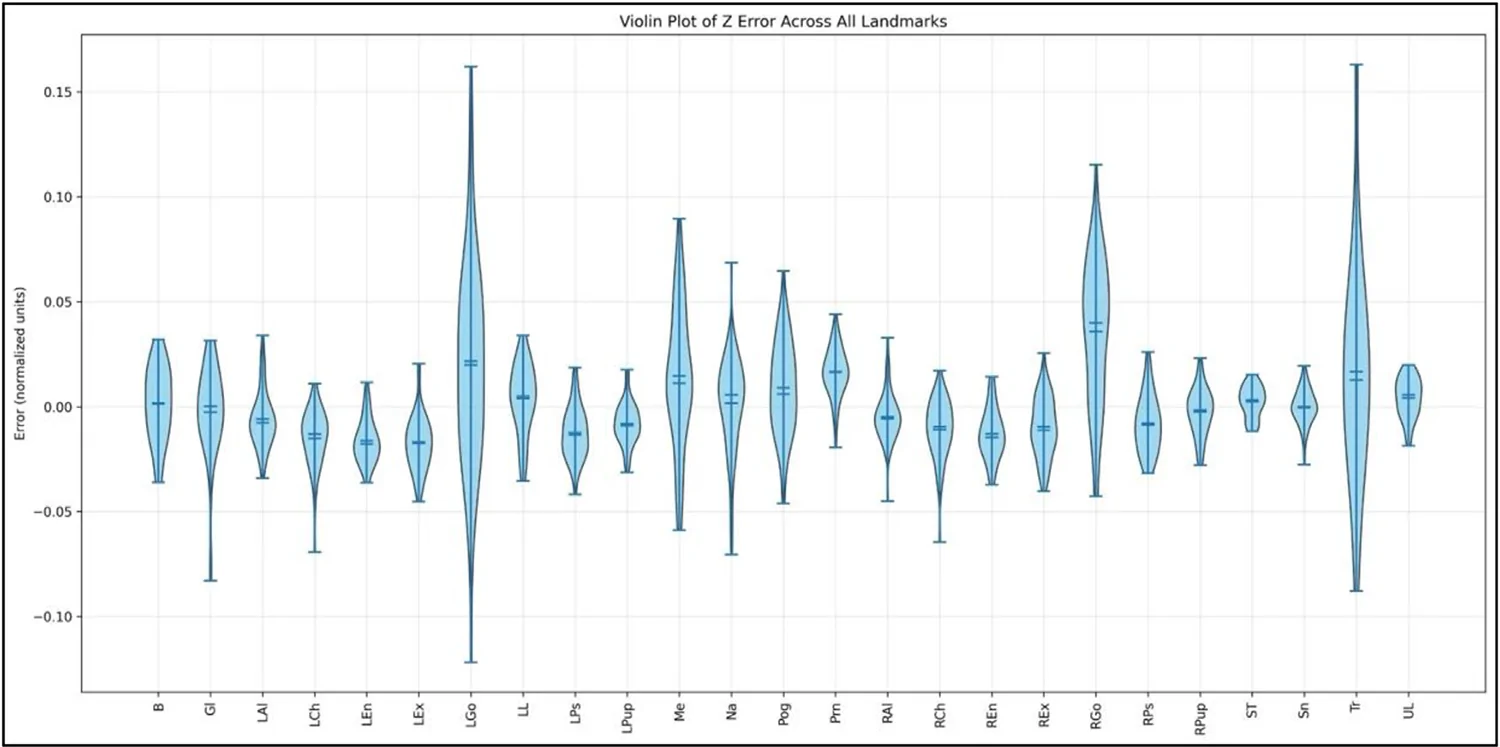
Violin Plots of Anterior-Posterior (Z-axis) Error by Landmark.

**Fig. 7.**
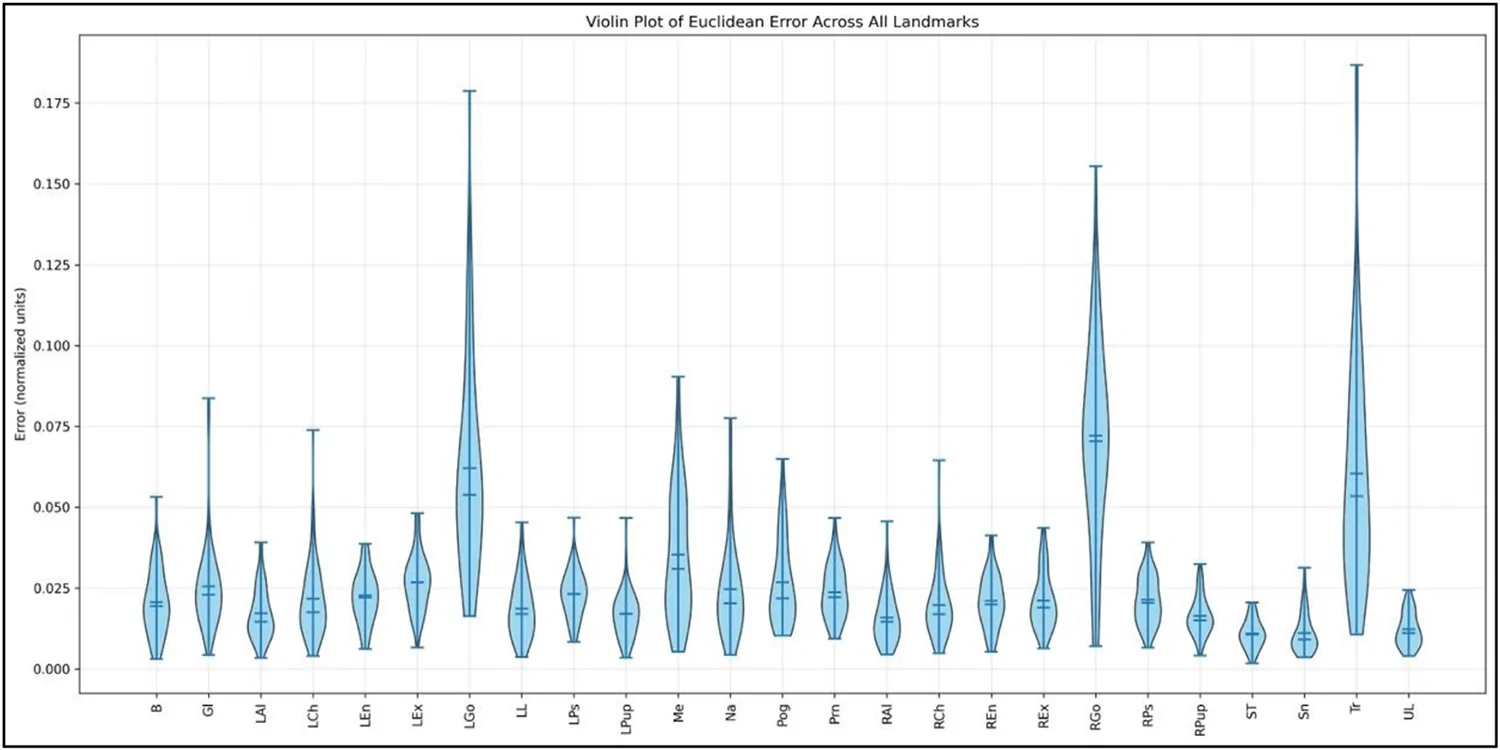
Violin Plots of Euclidean Distances Error by Landmark.

**Fig. 8.**
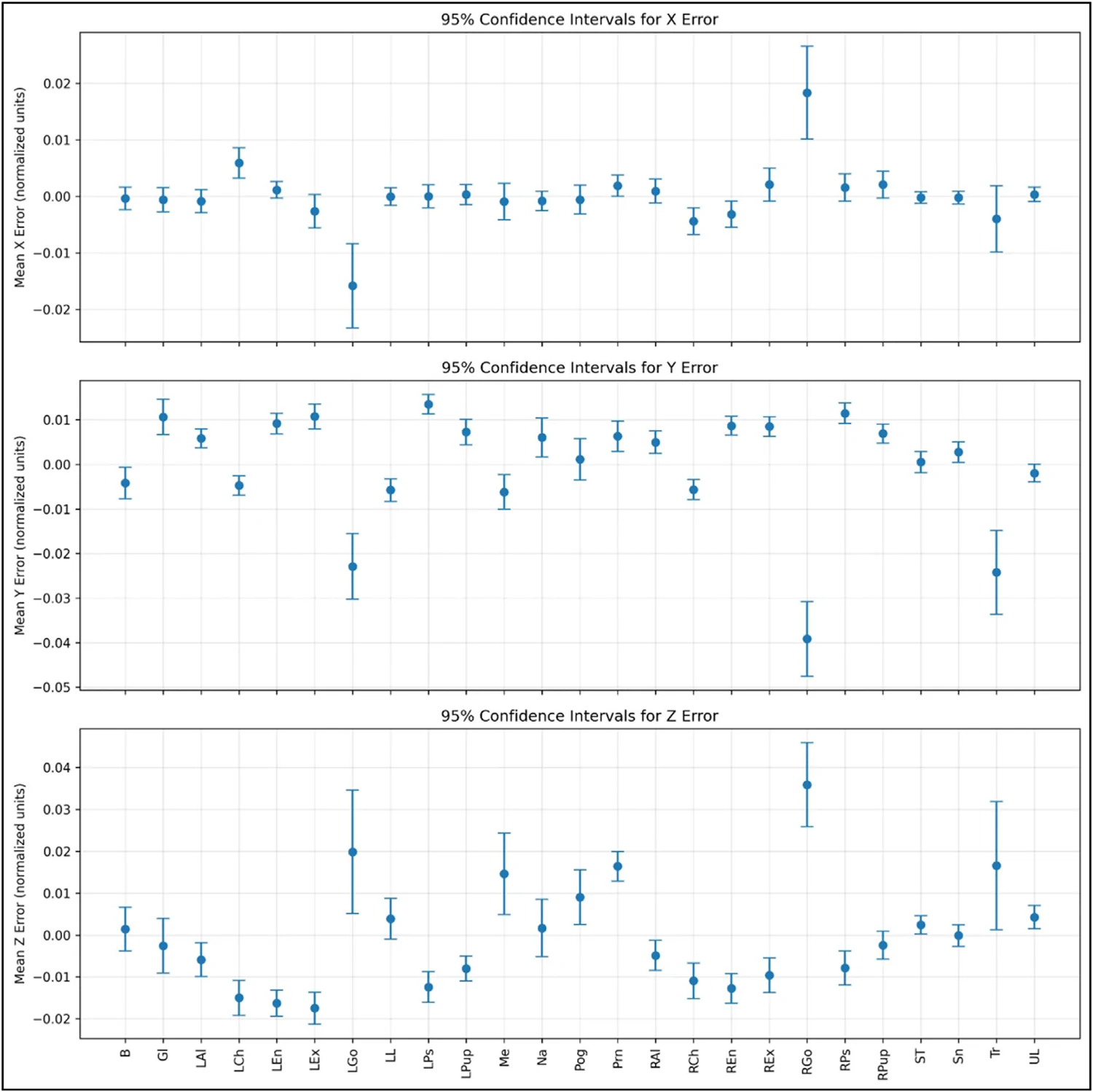
95 % Confidence Intervals for Mean Landmark Localization Error Across X, Y, and Z Axes.

**Fig. 9.**
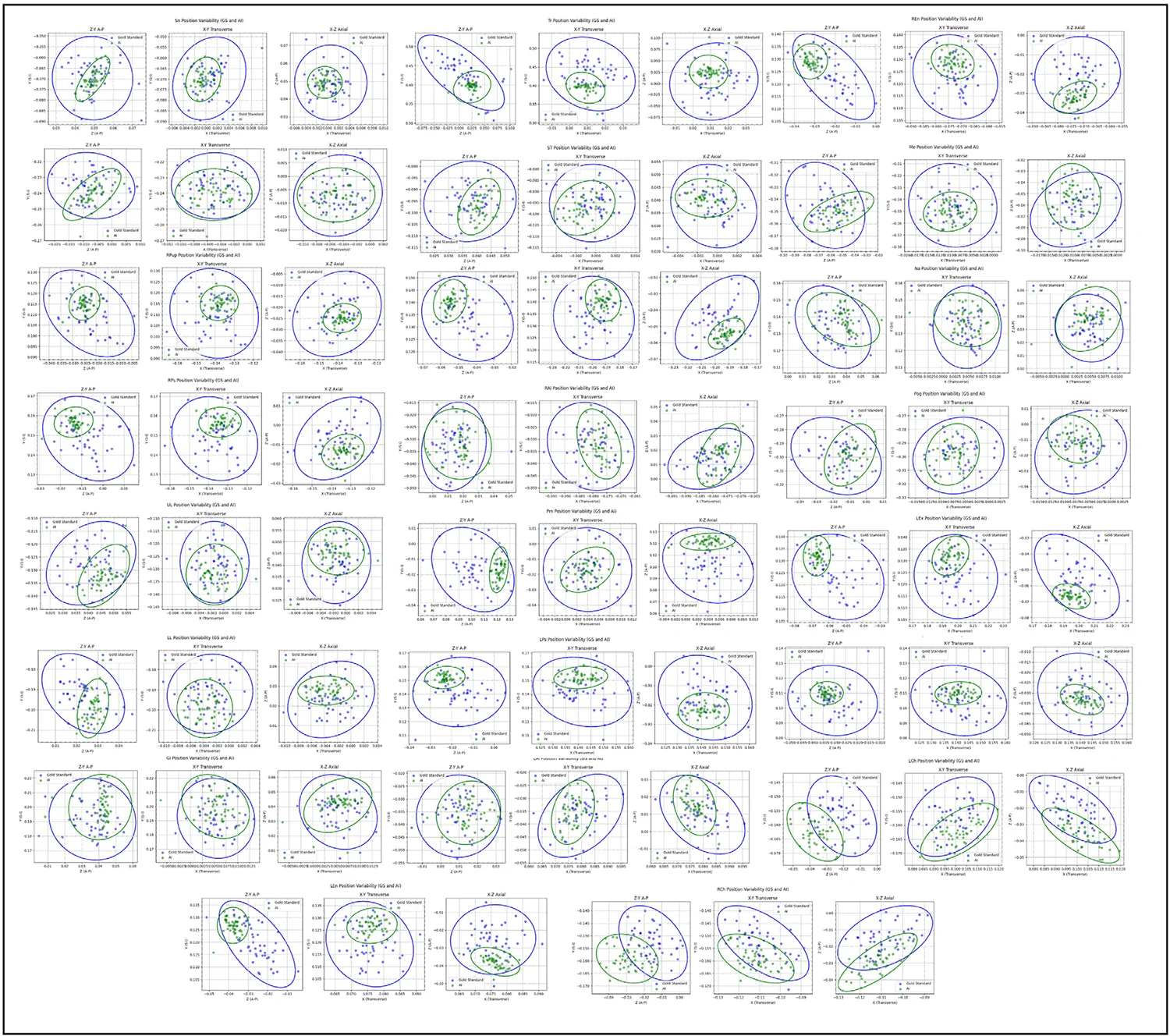
Root Mean Square Error (RMSE) of AI-based 3D Facial Landmark Reconstruction by Landmark (excluding LGo and RGo).

**Table 1:** Anatomical Landmarks and Definitions.

Abbreviation	Full name	Facial definition
Tr	Trichion	Midpoint on the hairline at the forehead.
Gl	Glabella	Most prominent point in the midline of the forehead, located between the eyebrows.
Na	Nasion	Depression at the junction of the frontal bone and nasal bones, at the root of the nose.
LPs	Left palpebrale superius	Midpoint of the upper eyelid margin of the left eye, in primary gaze position.
RPs	Right palpebrale superius	Midpoint of the upper eyelid margin of the right eye, in primary gaze position.
LPup	Left palpebrale inferius	Midpoint of the lower eyelid margin of the left eye.
RPup	Right palpebrale inferius	Midpoint of the lower eyelid margin of the right eye.
LEx	Left exocanthion	Lateral commissure where the upper and lower eyelids meet on the left eye.
REx	Right exocanthion	Lateral commissure where the upper and lower eyelids meet on the right eye.
LEn	Left endocanthion	Medial commissure where the upper and lower eyelids meet on the left eye.
REn	Right endocanthion	Medial commissure where the upper and lower eyelids meet on the right eye.
Prn	Pronasale	Most anterior and prominent point of the nasal tip.
LAl	Left alare	Most lateral point of the left nasal ala.
RAl	Right alare	Most lateral point of the right nasal ala.
Sn	Subnasale	Midline depression just below the base of the nose and above the upper lip.
ST	Soft tissue Point A (Subspinale)	Deepest midline depression of the upper lip, between Sn and UL; corresponds to hard tissue Point A in soft tissues.
UL	Upper lip	Most prominent point on the vermilion border of the upper lip.
LCh	Left cheilion	Left corner of the mouth where the upper and lower lips meet.
RCh	Right cheilion	Right corner of the mouth where the upper and lower lips meet.
LL	Lower lip	Most prominent point on the vermilion border of the lower lip.
B	Point B (supramental)	Midline depression between the lower lip and the soft tissue chin.
Pog	Pogonion	Most anterior and prominent point of the soft tissue chin.
Me	Menton	Lowest midline point on the soft tissue chin.
LGo	Left gonion	Most external point at the left mandibular angle (facial contour).
RGo	Right gonion	Most external point at the right mandibular angle (facial contour).
